# Heterotic quantitative trait loci analysis and genomic prediction of seedling biomass-related traits in maize triple testcross populations

**DOI:** 10.1186/s13007-021-00785-8

**Published:** 2021-07-30

**Authors:** Tifu Zhang, Lu Jiang, Long Ruan, Yiliang Qian, Shuaiqiang Liang, Feng Lin, Haiyan Lu, Huixue Dai, Han Zhao

**Affiliations:** 1 Jiangsu Provincial Key Laboratory of Agrobiology, Institute of Germplasm Resources and Biotechnology, Jiangsu Academy of Agricultural Sciences, Nanjing, 210014 China; 2 Jiangsu Provincial Key Laboratory of Agrobiology, Institute of Industrial Crops, Jiangsu Academy of Agricultural Sciences, Nanjing, 210014 China; 3 Institute of Tobacco, Anhui Academy of Agricultural Sciences, Hefei, 230001 China; 4 Nanjing Institute of Vegetable Sciences, Nanjing, 210042 China

**Keywords:** Seedling biomass-related traits, Heterotic quantitative trait loci, Genomic prediction, Triple testcross, Maize

## Abstract

**Background:**

Heterosis has been widely used in maize breeding. However, we know little about the heterotic quantitative trait loci and their roles in genomic prediction. In this study, we sought to identify heterotic quantitative trait loci for seedling biomass-related traits using triple testcross design and compare their prediction accuracies by fitting molecular markers and heterotic quantitative trait loci.

**Results:**

A triple testcross population comprised of 366 genotypes was constructed by crossing each of 122 intermated B73 × Mo17 genotypes with B73, Mo17, and B73 × Mo17. The mid-parent heterosis of seedling biomass-related traits involved in leaf length, leaf width, leaf area, and seedling dry weight displayed a large range, from less than 50 to ~ 150%. Relationships between heterosis of seedling biomass-related traits showed congruency with that between performances. Based on a linkage map comprised of 1631 markers, 14 augmented additive, two augmented dominance, and three dominance × additive epistatic quantitative trait loci for heterosis of seedling biomass-related traits were identified, with each individually explaining 4.1–20.5% of the phenotypic variation. All modes of gene action, i.e., additive, partially dominant, dominant, and overdominant modes were observed. In addition, ten additive × additive and six dominance × dominance epistatic interactions were identified. By implementing the general and special combining ability model, we found that prediction accuracy ranged from 0.29 for leaf length to 0.56 for leaf width. Different number of marker analysis showed that ~ 800 markers almost capture the largest prediction accuracies. When incorporating the heterotic quantitative trait loci into the model, we did not find the significant change of prediction accuracy, with only leaf length showing the marginal improvement by 1.7%.

**Conclusions:**

Our results demonstrated that the triple testcross design is suitable for detecting heterotic quantitative trait loci and evaluating the prediction accuracy. Seedling leaf width can be used as the representative trait for seedling prediction. The heterotic quantitative trait loci are not necessary for genomic prediction of seedling biomass-related traits.

**Supplementary Information:**

The online version contains supplementary material available at 10.1186/s13007-021-00785-8.

## Background

Hybrid breeding has successfully been used in maize over the last decades due to the direct utilization of heterosis, which refers to the agronomic performance superiority of heterozygous hybrids over corresponding homozygous parents [1]. The breeding process basically relies on empirical and time-consuming approaches. Preselection for candidates became essential for reducing significantly labor-intensive and economically prohibitive field-testing trials in multi-environments [2]. Genomic prediction, which could facilitate the rapid selection of superior genotypes, has emerged as a promising preselection tool for tackling this challenge [3]. Implementation of genomic prediction requires the training population with known phenotype and genotype to fit the prediction model, followed by predicting the genomic estimated breeding values of individuals in the testing populations, which are not phenotyped but are genotyped [4].

Recent studies have indicated the usefulness of various genomic prediction models involved in ridge regression best linear unbiased prediction (rrBLUP), genomic BLUP (GBLUP), and the general combining ability (GCA) model in prediction of yield-related traits and biomass-related traits (BRTs) in the harvested mature maize materials based on genomics, transcriptomics, and metabolites data [5–13]. Compared to the traditional pedigree approaches in plant breeding, predictive ability based on genomic prediction could be improved to different extents [14, 15]. The factors influencing predictive ability are usually marker density, the statistical model, population size and relationship, and heritability of the traits. In addition, population-wide linkage disequilibrium between quantitative trait loci (QTLs) and markers is also considered as information needed for the success of genomic prediction [16]. To determine the role of QTL in genomic prediction, QTLs can be incorporated into prediction models in an attempt to capture the comparable predictive ability. However, the effect of QTL in predictive ability is still controversial due to the observations that predictive ability with QTLs did not increase or increased slightly compared to that with random markers [10, 17, 18].

QTL mapping approaches have been extensively applied to identify genetic loci responsible for the complex traits and heterosis [19–24]. Among these approaches, the triple testcross (TTC) design, which allows three linear transformations *Z*_*1*_, *Z*_*2*_, and Z_*3*_ of the performance data of three populations involved in the F_2_ population or recombinant inbred line (RIL) population crossed with each parent and their F_1_, was used to test the significance of the epistatic effect of the QTL contributing to heterosis, with superiority in detection precision and efficiency [25]. As an elegant extension of the North Carolina experiment design III (NC III), TTC design has the advantage to produce unbiased estimation of genetic effects, assuming linkage and epistasis are absent [26]. Thus, the degree of dominance can be estimated from the ratio of unbiased dominance and additive variance [26].

Following the RIL-based TTC design, the heterotic QTLs of maize yield-related traits, including grain yield, number of kernels per plant, and kernel shape-related traits, were characterized [20, 27]. Apart from yield-related traits, one of the seedling BRTs, seedling dry weight (SDW) with ~ 40 days after sowing, was also focused on to decipher the gene action of heterotic QTLs using the TTC approach [20]. In total, four additive and five dominance heterotic QTLs for SDW with one-dimensional scan in the respective SUM (*Z*_*1*_) data set and DIFF (*Z*_*2*_) data set were detected, as well as five additive × additive (*aa*_*ij*_) and three dominance × dominance (*dd*_*ij*_) epistatic interactions with two-dimensional scans through the SUM data set and DIFF data set, respectively [20]. However, Melchinger et al. [28] defined two new types of heterotic gene effects, the augmented additive effect *a*_i_^*^, which includes the additive effect of QTL *i* (*a*_*i*_) minus half the sum of dominance × additive epistatic interactions (*da*_*ij*_), and the augmented dominance effect *d*_i_^*^, which includes the dominance effect of QTL i (*d*_*i*_) minus half the sum of additive × additive epistatic interactions with the genetic background irrespective of linkage, corresponding to the effects reflected in the SUM and DIFF data sets with one-dimensional scans, respectively. Thus, two-way marker interactions detected in the SUM and DIFF data sets should not be interpreted as the interactions of corresponding effects in one-dimensional scans. In fact, digenic epistatic interaction in the SUM data set comprised *aa*_*ij*_ and *dd*_*ij*_ interactions, while digenic epistatic interaction in the DIFF data set comprised *ad*_*ij*_ and *da*_*ij*_ interactions [26]. Unbiased estimates of *aa*_*ij*_ and *dd*_*ij*_ epistasis can be obtained with two-way analysis of variance (ANOVA) of *H*_*3*_ (the performance of progenies of F_2_ or RIL crossed with F_1_) and *Z*_*3*_ according to the genetic expectations of QTL effects for TTC designs reported by Melchinger et al. [26]. Hence, genetic effects, especially epistatic effects, contributing to heterosis of maize BRTs have not been comprehensively characterized.

In this study, TTC populations were constructed to identify heterotic QTLs and the epistatic interactions contributing to seedling BRTs. Predictive ability of seedling BRTs and the impact of heterotic QTLs on the predictive ability were evaluated.

## Methods

### Plant material and agronomic data

Intermated B73 × Mo17(IBM) population consisted of 122 RIL genotypes [29] were used as base population for creating TTC populations. Three testcross (TC) populations obtained by crossing each of 122 IBM genotypes with three testers (B73, Mo17, and B73 × Mo17) based on the TC design [25] were designated as TC(B), TC(M), and TC(F) populations, respectively. In total, 491 genotypes comprised B73, Mo17, B73 × Mo17, and 122 genotypes in each of four populations, IBMs, TC(B), TC(M), and TC(F), were included in this study. All of the genotypes were grown at the Experimental Station of the Jiangsu Academy of Agricultural Sciences with three blocks in a randomized complete block design in 2013. Each block comprised of 10 columns. Each column randomly arranged three testers and 49 genotypes from RIL and three TC populations as a single-row plot with 2.1 m length × 0.6 m width. The plants were finally thinned to eight per row. The seedling BRTs were measured at ~ 26 days after planting; leaf length (LL), leaf width (LW), and leaf area (LA) of the 3rd fully expanded leaf were determined by a portable leaf-scanning instrument (product ID LYM-B), while SDW was obtained from oven-dried aboveground plant samples collected within a row. Each trait per genotype within block was evaluated using at least three individual samples.

### Data analysis

B73 × Mo17, TC(B), TC(M), and their corresponding parents were subject to heterosis assessment by relative mid-parent heterosis (MPH), which was calculated using the equation MPH (%) = (F_1_ − MP)/MP × 100, where MP indicates the average value of the parents. For each trait, ANOVA was conducted on best linear unbiased estimates (BLUEs) of each genotype in TC populations across blocks. A *t*-test and Pearson’s correlation based on BLUEs were further used for significance testing and relationship analysis. To evaluate the heritability of seedling BRTs, $\sigma_{{{\text{GCA}}^{{\it\text{M}}} }}^{2} , \sigma_{{{\text{GCA}}^{{\it\text{P}}} }}^{2}$, and $\sigma_{{{\text{SCA}}}}^{2}$ of each trait was first determined by the R package ‘sommer’v4.1.2 [30], where $\sigma_{{{\text{GCA}}^{{\it\text{M}}} }}^{2} , \sigma_{{{\text{GCA}}^{{\it \text{P}}} }}^{2}$, and $\sigma_{{{\text{SCA}}}}^{2}$ correspond to maternal GCA, paternal GCA, and special combining ability (SCA) variance component, respectively. Then, narrow-sense heritability was defined as ${\it\text{h}}^{2} = \left( {\sigma_{{{\text{GCA}}^{{\it\text{M}}} }}^{2} + \sigma_{{{\text{GCA}}^{{\it\text{P}}} }}^{2} } \right) / \left( {\sigma_{{{\text{GCA}}^{{\it\text{M}}} }}^{2} + \sigma_{{{\text{GCA}}^{{\it\text{P }}} }}^{2} + \sigma_{{\text{SCA }}}^{2} + \sigma_{{\text{R}}}^{2} } \right)$, and broad-sense heritability was defined as ${\it\text{H}}^{2} = \left( {\sigma_{{{\text{GCA}}^{{\it\text{M}}} }}^{2} + \sigma_{{{\text{GCA}}^{{\it\text{P}}} }}^{2} + \sigma_{{{\text{SCA}}}}^{2} } \right) / \left( {\sigma_{{{\text{GCA}}^{{\it\text{M}}} }}^{2} + \sigma_{{{\text{GCA}}^{{\it\text{P }}} }}^{2} + \sigma_{{\text{SCA }}}^{2} + \sigma_{{\text{R}}}^{2} } \right)$ [31], in which $\sigma_{{\text{R}}}^{2}$ is the residual error variance. For any given trait, if the progenies of TC(B), TC(M), and TC(F) were denoted as *H*_*1i*_, *H*_*2i*_, and *H*_*3i*_ (*i* = 1–122), respectively, the following linear transformations of *Z*_*1i*_ = (*H*_*1i*_ + *H*_*2i*_)/2, *Z*_*2i*_ = *H*_*1i*_  − *H*_*2i*_, and *Z*_*3i*_ = *H*_*1i*_ + *H*_*2i*_ – 2*H*_*3i*_ (based on BLUEs of *H*_*1i*_, *H*_*2i*_, and *H*_*3i*_) were used for augmented additive, augmented dominance, and epistasis heterotic QTL detection [28].

### QTL mapping

A total of 1631 markers, including 142 single nucleotide variations, 93 insertion/deletion, 57 presence/absence variations [27], and other public markers were used for linkage map construction using IciMapping [32]. This software was further used to implement QTL mapping via the inclusive composite interval mapping algorithm, which can avoid biased estimation of QTL effects resulting from the commonly used method, composite interval mapping, due to the potential absorption of the QTL effect in the current testing interval by the background marker variables. In one-dimensional scans, LOD scores were evaluated based on 1000 permutations with the default probability of 0.001 and a default walk step of 1 cM. In two-dimensional scans, the LOD scores were evaluated based on 1000 permutations with the default probability of 0.0001 and a default walk step of 5.0 cM. The gene mode of a QTL evaluated by the dominance ratio degree *|d*_i_^*^/*a*_i_^*^| was classified into four groups: additive (|*d*_i_^*^/a_i_^*^|< 0.2), partially dominant (0.2 ≤|*d*_i_^*^/a_i_^*^|< 0.8), dominant (0.8 ≤|*d*_i_^*^/a_i_^*^|< 1.2), or overdominant (|*d*_i_^*^/a_i_^*^|≥ 1.2) [20].

### Genomic prediction

The performance of TC(B) and TC(M) progenies was used as the response variable in the genomic prediction model. The genotypes of these two populations were derived from the corresponding genotypes of IBM individuals and either B73 or Mo17 defined by 1631 markers. For missing genotypes, the mean value of the marker was assigned by the R package ‘rrBLUP’ v4.6 [33]. The universal model for GCA and SCA effects was employed for prediction [8]:

$${\text{y}} = \mu + {\text{Z}}_{{\it\text{M}}} {\text{g}}_{{\it\text{M}}} + {\text{Z}}_{{\it\text{P}}} {\text{g}}_{{\it\text{P}}} + {\text{Z}}_{{\it\text{S}}} {\text{s}} + \in$$,

with

$${\text{g}}_{{\it\text{M}}} \sim {\text{N}}\left( {0, {\text{G}}_{{\it\text{M}}} \sigma_{{{\text{GCA}}^{{\it\text{M}}} }}^{2} } \right),\;{\text{g}}_{{\it\text{P}}} \sim {\text{N}}\left( {0, {\text{G}}_{{\it\text{P}}} \sigma_{{{\text{GCA}}^{{\it\text{P}}} }}^{2} } \right),\;{\text{s}} \sim {\text{N}}\left( {0, {\text{G}}_{{\it\text{S}}} \sigma_{{{\text{SCA}}}}^{2} } \right)$$

where y is the vector of the observed averaged hybrid performance across three blocks, *μ* is the fixed model intercept, ${\text{Z}}_{{\it\text{M}}}$, ${\text{Z}}_{{\it\text{P}}}$, and ${\text{Z}}_{{\it\text{S}}}$ correspond to the design matrix related to the random GCA effect (${\text{g}}_{{\it\text{M}}}$) of maternal lines, random GCA effect (${\text{g}}_{{\it\text{P}}}$) of paternal lines, and SCA effect (${\text{s}}$), respectively. The genomic relationship matrix can be described as follows [8]:

$${{\rm{G}}_{\rm\it{M}}} = \frac{1}{{\rm\it{N}}}{{\rm{W}}_{\rm\it{M}}}{\rm{W}}_{\rm\it{M}}^{\rm{\text{T}}},\;{{\rm{G}}_{\rm\it{P}}} = \frac{1}{{\rm\it{N}}}{{\rm{W}}_{\rm\it{P}}}{\rm{W}}_{\rm\it{P}}^{\rm{\text{T}}}$$

where *N* denotes the marker number, ${\text{W}}_{{\it\text{M}}}$ and ${\text{W}}_{{\it\text{P}}}$ represent the standardized marker matrix for respective maternal lines and paternal lines, and ${\text{W}}_{{\it\text{M}}}^{{ \text{T}}}$ and ${\text{W}}_{{\it\text{P}}}^{\text{T}}$ represent transposed ${\text{W}}_{{\it\text{M}}}$ and ${\text{W}}_{{\it\text{P}}}$, respectively. The rows and columns of the matrix are determined by the parent number and marker number, respectively. Each matrix column is centered and standardized to unit variance.${\text{G}}_{{\it\text{S}}}$ was produced by multiplying respective elements in ${\text{G}}_{{\it\text{M}}}$ and ${\text{G}}_{{\it\text{P}}}$ [34].

The prediction was carried out in the R package ‘sommer’ v4.1.2 [30]. To achieve the best prediction, the block effect and spatial effect fit by spl2D() function for rows and columns in the field were also considered. A five-fold cross-validation scheme with 1000 runs was employed to obtain unbiased estimates of predictive ability, also referred to as prediction accuracy. For each run, 80% hybrids randomly selected as the training set were used to calibrate the prediction model, followed by the prediction of the validation set, comprised of the remaining 20% hybrids, and then Pearson correlation was calculated between observed and predicted hybrid performance in the validation set as the prediction accuracy.

## Results

### Phenotypic summary and heterosis

**Fig. 1:**
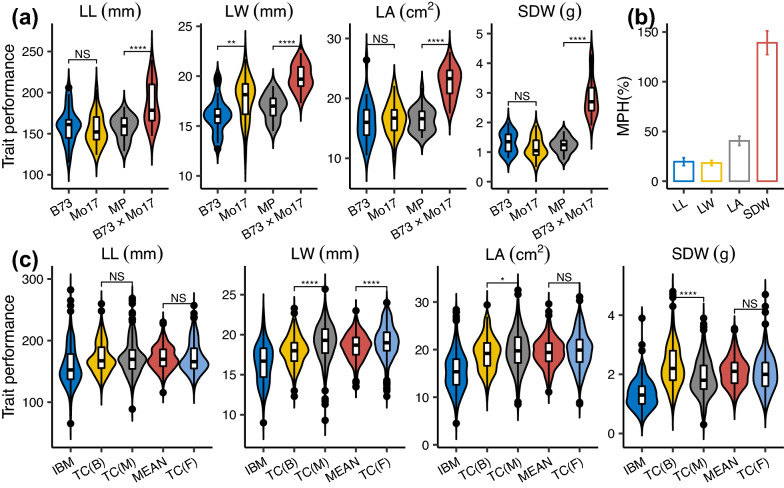
Performance and heterosis of seedling BRTs. **a** Violin plot of seedling BRTs in parental lines and B73 × Mo17. Double asterisks (**) and four asterisks (****) indicate significant differences at the *p* < 0.01 and *p* < 0.0001 levels, respectively, according to the *t*-test. NS represents not significant. **b** Heterosis levels of seedling BRTs. **c** Violin plot of seedling BRTs in IBM and three TC populations. Group MEAN on the X-axis represents the mean values of corresponding progenies derived from the same IBM genotype in TC(B) and TC(M) populations. A single asterisk (*) indicates a significant difference at the *p* < 0.05 level according to the *t*-test

ANOVA based on three TC populations revealed a highly significant difference among TC populations (Additional file 1: Table S1), implying the suitability of TTC design. Heritability (${\it\text{h}}^{{2}}$) estimates for seedling BRTs were low, ranging from 0.10 (LL) to 0.44 (SDW), as were ${\it\text{H}}^{{2}}$ (Additional file 1: Table S2). Statistical analysis between parental lines showed that B73 had higher trait values in LL and SDW but lower trait values in LW and LA, of which only LW differed significantly (Fig. 1a). When comparing MP with B73 × Mo17, we found that all of the BRTs in B73 × Mo17 were significantly greater than those in MP (Fig. 1a). This higher observation for seedling BRTs was also reflected by MPH (Fig. 1b). Among these traits, SDW had the highest MPH (~ 150%), whereas the three other traits had lower MPH, less than 50% (Fig. 1b). With respect to the performance of seedling BRTs in populations, the trait values of three TC populations were all higher than IBM, as expected (Fig. 1c). Significant differences between TC(B) and TC(M) were observed for seedling BRTs except LL, which confirmed that B73 alleles were beneficial for SDW and Mo17 alleles were beneficial for LW as well as LA (Fig. 1c). Moreover, LW exhibited a significant difference compared with the mean of TC(B) and TC(M) with TC(F) (Fig. 1c).

**Fig. 2:**
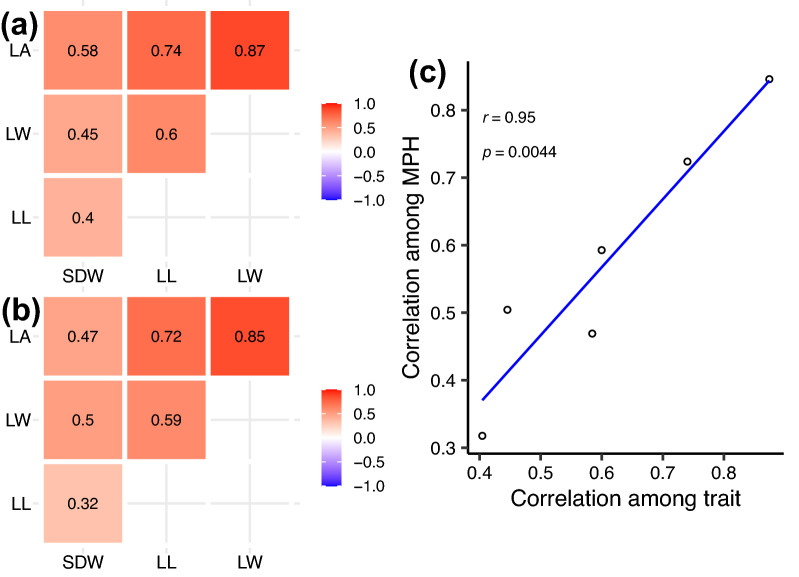
Correlation of performance and heterosis of seedling BRTs. Correlations between trait BLUEs (**a**) and between heterosis (**b**) of seedling BRTs. The number represents the Pearson’s *r* with *p* < 0.01 and is ordered using hierarchical clustering. **c** Scatter plot of the correlation of performance and heterosis of seedling BRTs

To understand the relationships between seedling BRTs, Pearson’s correlations were estimated based on BLUEs of TC populations. The strongest significant correlation occurred between LW and LA (*r* = 0.87), whereas the lowest significant correlation was found between LL and SDW (*r* = 0.40) (Fig. 2a). Heterosis of seedling BRTs displayed the identical relationship tendency, with Pearson’s correlation coefficient ranging from 0.32 between LL and SDW to 0.85 between LW and LA (Fig. 2b). This trend was further supported by the extremely high correlation between the correlation of MPH and performance of seedling BRTs (Fig. 2c). To determine the contribution of heterozygosity to the seedling BRTs, 1631 markers were used to derive the heterozygosity of TC(B) and TC(M) genotypes. The low correlations between heterozygosity and the traits indicated that the role of heterozygosity can be ignored in shaping the performance and heterosis of seedling BRTs (Additional file 2: Fig. S1).

### Identification of heterotic QTLs

A linkage map covering 6943.84 cM was constructed by 1631 markers, with an average interval distance of 4.28 cM between adjacent markers (Additional file 1: Table S3). For each chromosome, the genetic distance between adjacent markers ranged from 3.58 to 4.78 cM (Additional file 1: Table S3).

In total, 19 heterotic QTLs for seedling BRTs distributed on six chromosomes were detected with three *Z* transformations in one-dimensional scan in which 14 augmented additive QTLs were in *Z*_*1*_, two augmented dominance QTLs were in *Z*_*2*_, and three dominance × additive epistatic QTLs were in *Z*_*3*_ (Table 1). The phenotypic variation explained due to a single QTL ranged from 4.1 to 20.5%; both occurred in augmented additive QTLs for LA (Table 1).

**Table 1:** Heterotic QTLs detected in *Z*_*1*_, *Z*_*2*_, and *Z*_*3*_ for seedling BRTs

BRTs	QTL	Chr.	Marker interval	*Z*_*1*_			*Z*_*2*_			|*d*_i_^*^/*a*_i_^*^|	*Z*_*3*_		
				LOD	*a*_i_^*^	*R*^*2*^(%)	LOD	*d*_i_^*^	*R*^*2*^(%)		LOD	*da*_*i*_	*R*^*2*^(%)
LL	*qLL4a*	4	InDel70-umc1142	2.9	4.1	7.6				PD			
	*qLL7a*	7	umc1015-umc1713	4.7	− 5.1	11.6				PD			
	*qLL7b*	7	umc1015-umc1713							PD	2.8	-9.7	10.5
LW	*qLW2a*	2	bnlg1327-bnlg2277	3.1	0.4	7.1				PD			
	*qLW4a*	4	InDel68-S4_69842356	4.2	0.5	10.4				PD			
	*qLW7a*	7	umc1412-InDel107	3.0	0.4	8.3				D			
	*qLW7b*	7	umc2160-umc1159	3.1	− 0.4	7.2				A			
	*qLW9a*	9	umc1586-S9_24472332	2.6	0.5	10.2				A			
LA	*qLA4a*	4	InDel68-S4_69842356	10.7	1.4	20.5				A			
	*qLA4b*	4	umc2061-umc1969	3.7	− 0.7	6.1				A			
	*qLA7a*	7	umc2160-umc1159	3.1	− 0.7	5.1				PD			
	*qLA9a*	9	umc1271-asg63a	2.6	0.6	4.1				PD			
	*qLA10a*	10	bnlg1712-phi050	5.8	0.9	9.7				PD			
	*qLA4c*	4	umc1164-bx4							D	3.3	1.9	11.4
SDW	*qSDW4a*	4	npi270-php20071	2.9	− 0.1	8.4				PD			
	*qSDW7a*	7	S7_21278179-umc1978	3.9	− 0.1	11.5				PD			
	*qSDW5a*	5	ufg49-umc1315				2.7	0.2	8.1	D			
	*qSDW10a*	10	umc2122-umc1993				2.8	0.2	10.1	OD			
	*qSDW4b*	4	PAV5-umc1669							OD	3.3	0.5	12.0

*LL* leaf length, *LW* leaf width, *LA* leaf area, *SDW*: seedling dry weight, *Chr* chromosome ID, *LOD* logarithm of the odds, *a*_i_^*^ the augmented additive effect, *R*^*2*^ phenotypic variation explained by individual QTL effects, *d*_i_^*^ the augmented dominance effect, *da*_*i*_ dominance × additive epistatic effect

For LL, three QTLs located on chromosomes 4 and 7 were found in *Z*_*1*_ and *Z*_*3*_. Two augmented additive QTLs explained 7.6 and 11.6%, respectively, of the variation in LL. Both QTLs were partially dominant with effects for *Z*_*1*_ being 4.1 and − 5.1 respectively, indicating alleles from B73 and Mo17 both contributed to increasing LL (Table 1).

For LW, five QTLs mapped on chromosomes 2, 4, 7, and 9 were detected, all in *Z*_*1*_. Individual QTL accounted for 7.1–10.4% of variation in LW. Additive, partially dominant, and dominant gene modes were observed for these augmented additive QTLs (Table 1). Among these QTLs, four showed positive effects, and one showed a negative effect, indicating alleles increasing this trait are provided by both B73 and Mo17.

For LA, six QTLs were identified, with five in *Z*_*1*_ and one in *Z*_*3*_. Five augmented additive QTLs were distributed on four chromosomes, with *qLA4a* on chromosome 4 owing the largest contribution to the phenotypic variation of LA (20.5%), colocalized with *qLW4a*, compared with *qLA7a* on chromosome 7, with the small contribution to the phenotypic variation of LA (5.1%), colocalized with *qLW7b*. Two QTL regions shared by LW and LA suggest that these two genetic loci may contribute to LA and LW simultaneously although the gene action of each locus affecting each trait differently (Table 1). In total, augmented additive QTLs accounted for 45.5% of phenotypic variation, whereas phenotypic variation due to the dominance × additive epistatic effect was 11.4%.

For SDW, five QTLs were revealed, with two in *Z*_*1*_, two in *Z2*, and one in *Z*_*3*_. Two augmented additive QTLs on chromosomes 4 and 7 accounted for 8.4 and 11.5%, respectively, of phenotypic variation. The effects of both QTLs were negative, indicating alleles donated from Mo17 led to improvement of SDW. For two augmented dominance QTLs located on chromosomes 5 and 10, 8.1 and 10.1% of the respective phenotypic variation was accounted for by each locus with different gene action (Table 1). For one epistatic QTL on chromosome 4, which was closely linked to epistatic loci *qLA4c* for LA, the phenotypic variation due to the dominance × additive epistatic effect reached 12.0%.

Digenic epistatic interactions for seedling BRTs were detected in *H*_*3*_ and *Z*_*3*_ with two-dimensional scans. In *H*_*3*_, ten marker pairs of additive × additive epistatic interaction were identified for seedling BRTs (Table 2). Among these marker pairs, five interactions were for LL with an additive × additive epistatic effect (three were negative and two were positive) explaining between 9.6 and 13.2% of phenotypic variation, while three interactions for LW, LA and DW, with 9.8, 17.3, and 18.6% of the respective phenotypic variation explained, due to additive × additive epistatic effects. In *Z*_*3*_, six marker pairs of dominance × dominance epistatic interactions were detected across all of the seedling BRTs (Table 2). The largest proportion of phenotypic variation explained by the dominance × dominance epistatic effect reached 23.6% for SDW, while the smallest was 9.4% for LL and SDW. In addition, all of the digenic interaction regions did not overlap with main-effect QTLs.

**Table 2:** Genetic intervals of digenic interaction for BRTs detected in *H*_*3*_ and *Z*_*3*_

BRTs		Chr.	Marker interval	Chr.	Marker interval	LOD	*a*_*i*_	*a*_*j*_	*aa*_*ij*_	*R*^*2*^(%)
LL	*H*_*3*_	3	npi425a-npi420	4	umc2139-umc52	5.3	− 1.6	− 0.3	− 6.9	10.1
		2	umc2030-umc1259	4	psr144b-umc1943	5.2	− 2.0	− 1.7	− 7.0	9.6
		4	agrr301-bnlg490	5	S5_212169465-InDel90	5.5	− 2.9	1.6	− 7.0	9.6
		4	phi295450-InDel63	8	umc1913-csu329	5.9	− 2.3	− 0.4	7.3	13.2
		4	InDel78-S4_240467004	9	mmp131-asg44	5.3	− 1.8	− 0.7	6.5	9.7
	*Z*_*3*_	8	mmp166-npi585a	9	umc1691-umc1271	5.2	2.8	5.8	− 12.6	10.1
		9	umc1570-lim99b	9	isu111b-csu471	5.3	6.7	− 1.1	13.1	9.4
LW	*H*_*3*_	2	umc2030-umc1259	2	npi287a-umc44b	5.8	0.1	0.1	0.7	9.8
		5	mmp169-php20566	5	S5_1531780-PAV58	5.0	0.0	0.0	− 0.6	8.4
		3	npi420-InDel62	10	asg19b-csu48	5.2	0.2	0.0	0.8	14.0
	*Z*_*3*_	3	mmc0022-InDel58	3	npi420-InDel62	6.0	1.1	− 0.7	1.8	19.7
LA	*H*_*3*_	1	umc1177-tub1	6	rz143a-umc85a	5.2	0.3	0.3	− 1.1	17.3
	*Z*_*3*_	5	S5_1531780-PAV58	8	umc1316-bnl12.30a	5.1	1.0	0.7	− 2.2	18.3
SDW	*H*_*3*_	1	lim122-cdo1387b	6	umc85a-isu085a	5.1	0.0	0.0	− 0.2	18.6
	*Z*_*3*_	1	asg30b-umc1169	8	cdo460-mmp57	5.5	− 0.1	0.1	0.4	9.4
		4	PAV42-PAV5	9	PAV93-PAV30	6.3	0.4	0.4	− 0.6	23.6

*LL* leaf length, *LW* leaf width, *LA* leaf area, *SDW* seedling dry weight, *Chr* chromosome ID, *LOD* logarithm of the odds, *a*_*i*_ the main effect of locus *i*, *a*_*j*_ the main effect of locus *j*, *aa*_*ij*_ the epistatic effect between loci *i* and *j*, *R*^*2*^ trait variation explained by the epistatic QTL effect

### Genomic prediction of seedling BRTs

**Fig. 3:**
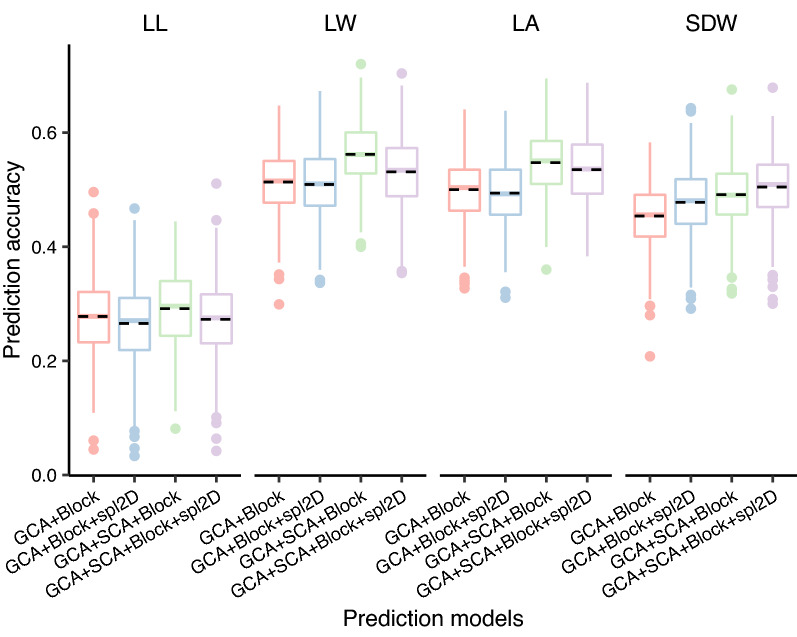
Comparison among prediction models. The black dashed line represents the mean of prediction accuracies with 1000 iterations

**Fig. 4:**
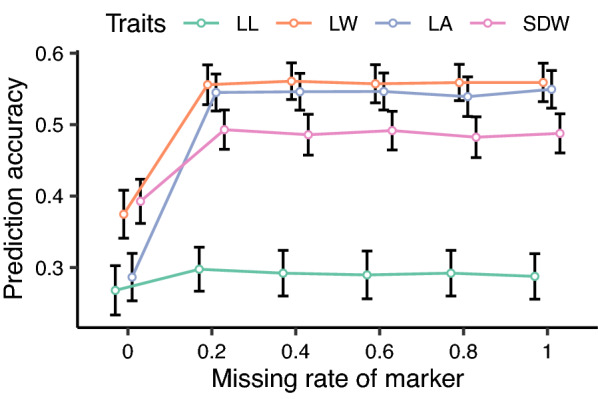
Prediction accuracies of seedling BRTs based on different imputed marker groups. Prediction accuracies are expressed as the mean ± standard deviation of 1000 cross-validations. Numerals on the X-axis represent the maximum missing rate of markers for each group. The marker with a missing rate (proportion of individuals with missing genotype at the given marker locus) lower than the maximum missing rate would be imputed, and a marker higher than the maximum missing rate would be excluded from that group. The number 0 indicates that the group is comprised of non-missing markers, and 1 indicates that the group is comprised of all of 1631 markers. For each group, the error bars were separated to avoid overlapping with each other

In total, 244 heterozygous genotypes of the TC(B) and TC(M) population with observations in three blocks were used for genomic prediction for seedling BRTs based on 1631 markers. Comparisons among models if incorporating SCA and/or spatial effects revealed that the universal model for GCA and SCA effects coupling with block effect could capture the best predictions across seedling BRTs, and therefore used in subsequent analyses (Fig. 3). To test the influence of imputed markers on prediction accuracy, six marker groups comprising imputed markers were selected. Each group with the maximum missing rate 0, 0.2, 0.4, 0.6, 0.8, and 1 includes a total of 30, 1394, 1623, 1629, 1631, and 1631 markers used for prediction. Prediction accuracies for all of the seedling BRTs showed negligible discrepancy among marker groups when the maximum missing rate exceeded 0.2 (Fig. 4), indicating all 1631 markers could be used for predictions, regardless of being imputed. Overall, among seedling BRTs, LL had the lowest predictive ability (0.29), whereas the three other traits had modest predictive ability ranging from 0.49 for SDW to 0.56 for LW. To evaluate the predictive effect of different numbers of 1631 markers, we randomly selected seven marker groups with the marker number between 1 and 1600, with five repeats for each group. The prediction results demonstrated a notable plateau of prediction accuracy could be achieved by 400 markers for all of the seedling BRTs (Additional file 2: Fig. S2). When the markers were extended to 800, the prediction accuracies remained stable and were almost not enhanced when more markers were used.

**Fig. 5:**
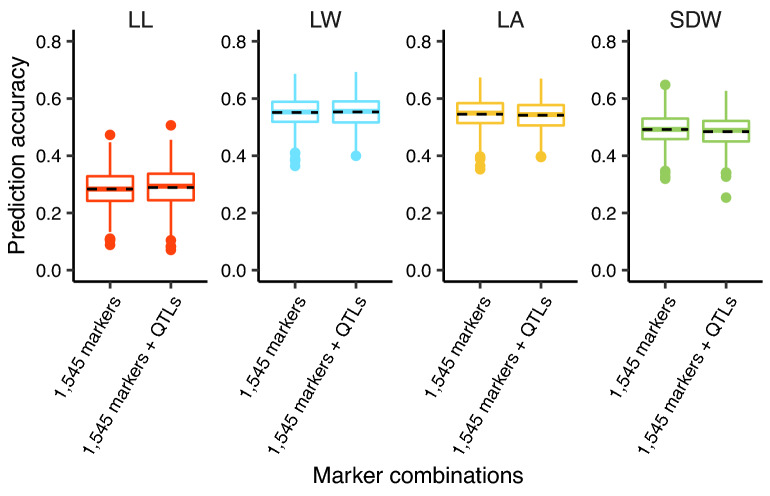
Comparison among prediction accuracies based on markers integrating corresponding QTLs of seedling BRTs. The black dashed line represents the mean of prediction accuracies with 1000 iterations

The prediction model was further fit with marker-enclosed augmented additive QTLs, augmented dominance QTLs, and epistatic QTLs for each seedling BRT. Compared with 1545 markers not related to determination of QTL intervals, integration of any type of QTLs does not return significant improvement of prediction accuracies, of which a subtle increase by 1.7% was found in LL (Fig. 5).

## Discussion

A number of QTLs related to BRTs, including plant height, leaf area, and plant weight aboveground at the seedling or silking stage under low nitrogen and phosphorus, drought, or normal conditions, have been revealed in maize [35–37]. However, few studies have been conducted on heterotic QTLs involved in BRTs. Using RIL-based NC III, six and eight environmentally stable main heterotic QTLs of PH and EH were identified from *Z*_*1*_ and *Z*_*2*_, respectively [19]. Using the RIL-based TTC design, a total of 12 main heterotic QTLs related to SDW (~ 40 days after sowing) were detected, which accounted for 13.6 and 31.3% of the variation due to augmented additive and dominance effects, respectively [20]. In our study, we characterized comprehensively heterotic QTLs of maize seedling BRTs. Among these, 14 were augmented additive and two were augmented dominant (Table 1). All four modes of gene action, i.e., additive, partially dominant, dominant, and overdominant, were observed for these loci. The two overdominant loci were detected only in SDW in which one exhibited an augmented dominance effect and the other exhibited a dominance × additive effect (Table 1). This might be ascribed to the high MPH (~ 150%) of SDW because complete dominance alone might not be sufficient to explain such high heterosis and overdominance and/or epistasis should be taken into account in terms of the contribution to heterosis when heterosis is more than 100%. Moreover, the high level of heterosis likely increased the number of augmented dominance QTLs detected, partly accounting for the fact that augmented dominance QTLs were identified in SDW but not in other seedling BRTs.

The epistatic effects have been demonstrated to play a prominent role in heterosis [19, 38, 39]. In our study, we revealed three QTLs with a dominance × additive effect in one-dimensional scan for *Z*_3_, reflecting QTL × genetic background interactions, as well as ten marker pairs of the additive × additive epistatic interaction in *H*_*3*_ and six marker pairs of the dominance × dominance epistatic interaction in *Z*_*3*_ with a two-dimensional scan. Among these epistatic interactions, six additive × additive epistatic interaction displayed negative directions. According to the expression of heterosis MPH = [*d*] – 0.5[*aa*] [28], these negative additive × additive epistatic effects could contribute positively to heterosis. Hence, even small epistatic interactions can be important for heterosis. The comprehensive identification of the main and interactive heterotic QTLs proven the high efficiency and robustness of RIL-based TTC approach.

QTL-associated markers with major effects are generally used to select individuals for simple traits by marker-assisted selection. In comparison, a genomic selection approach can incorporate all of the molecular markers of the whole genome regardless of marker effects to predict the performance of candidates for selection [4]. Some biomass-related traits in maize have been subject to genomic prediction. For dry matter yield of whole-plant aboveground biomass in the harvested materials, the prediction accuracy displayed a considerably large range between 0.5 and 0.8 for most of the various predictors and predictor combinations [8]. In contrast, the prediction accuracy of SDW in our analysis was less than 0.5. The discrepancy may be caused by the change in heritability of SDW from early (0.49) to later (0.82) developmental stages due to the fact that high heritability can lead to an increase in prediction accuracy [40]. Moreover, this is also supported by the consistent tendency between prediction accuracy and heritability among seedling BRTs. In addition to heritability, marker density has been demonstrated to substantially influence prediction accuracy before a prediction plateau where additional markers no longer had an effect on prediction accuracy improvement. The marker numbers that reached a plateau varied from dozens to thousands in numerous previous studies [6, 8, 41]. For seedling BRTs, 800 markers almost achieved the prediction plateau (Additional file 2: Fig. S2), indicating 1631 markers are sufficient to obtain the maximum prediction accuracy.

QTL-based genomic prediction in maize focused on the disease traits [10, 17, 18]. A slight improvement and decrease of prediction accuracy were both observed in BLUP models when incorporating disease resistance QTLs into genome-wide markers in those studies. In comparison with 1545 genome-wide markers, the inclusion of additional markers closely linked to major heterotic QTLs and epistatic interactions of seedling BRTs did not result in a dominant change of the prediction accuracy (Fig. 5). Three aspects could be considered to account for such results. The first explanation may be that the GCA and SCA model implemented belongs to the BLUP approach. This was evidenced indirectly in a stochastic simulation analysis in which the GBLUP model was found to have a constant accuracy irrespective of the number of QTLs under conditions of a given heritability and sample size [42]. The second explanation could be that the QTLs identified did not cover all of the loci, even those that were numerous with small effects, across the genome. For enhancing the prediction accuracy, one way is to incorporate all of the public markers detected significantly by either QTL mapping or genome-wide association analysis into the prediction model [43]. The third reason may be that the QTLs revealed are related to heterosis but not the performance per se of seedling BRTs. The heterotic QTLs might not favor the improvement of prediction for performance per se as a consequence of the subtle role of QTLs in the performance.

Apart from BLUP prediction models, Bayesian and machine learning models such as Random Forest and deep learning were also employed to explore the prediction [44]. In some cases, these methods could gain a better prediction performance [45–47]. However, due to the complexity of prediction, superior prediction models integrating the advantages of various methods remain to be developed in the future to fit QTLs.

## Conclusions

We precisely identified the main and epistatic heterotic QTLs of seedling biomass-related traits used triple testcross strategy. All four modes of gene action including additive, partially dominant, dominant, and overdominant modes were observed. We also found that prediction accuracy of seedling BRTs ranged from 0.29 for leaf length to 0.56 for leaf width and the incorporation of heterotic QTLs did not lead to the significant improvement of prediction accuracy. These findings demonstrated that the TTC design is suitable for detecting heterotic QTLs and evaluating the prediction accuracy. The heterotic QTLs are not necessary for genomic prediction of seedling BRTs.

## Supplementary Information

**Additional file 1**: **Table S1**. Analysis of variance for seedling BRTs in TC populations based on BLUEs. LL: leaf length, LW: leaf width, LA: leaf area, SDW: seedling dry weight. *, **, and *** represent significance at the 0.05, 0.01, and 0.001 levels, respectively. **Table S2**. Variance components and heritability of BRTs. LL: leaf length, LW: leaf width, LA: leaf area, SDW: seedling dry weight. **Table S3**. Information of linkage map constructed. Chr.: chromosome ID.

**Additional file 2**: **Fig. S1**. Scatter plot for performance and heterosis of seedling BRTs versus heterozygosity. (a) Relationships between performance of seedling BRTs with heterozygosity. (b) Relationships between MPH of seedling BRTs with heterozygosity. **Fig. S2**. Prediction accuracies of seedling BRTs based on different numbers of markers. Corresponding numbers of markers were sampled randomly from 1631 markers five times to represent five repeats of the marker group. The mean of the prediction accuracies with 1000 runs for each repeat was plotted.

## Data Availability

The datasets used and/or analyzed during the current study are available from the corresponding author on reasonable request.

## Abbreviations

ANOVA: Analysis of variance
BLUEs: Best linear unbiased estimates
BRTs: Biomass-related traits
GBLUP: Genomic best linear unbiased prediction
GCA: General combining ability
IBM: Intermated B73 × Mo17
LA: Leaf area
LL: Leaf length
LW: Leaf width
MPH: Mid-parent heterosis
NC III: North Carolina experiment design IIIQTLs: Quantitative trait loci
RIL: Recombinant inbred line
rrBLUP: Ridge regression best linear unbiased prediction
SCA: Special combining ability
SDW: Seedling dry weight
TTC: Triple testcross
