# Supplementary material for: Heterotic quantitative trait loci analysis and genomic prediction of seedling biomass-related traits in maize triple testcross populations
Source: Plant Methods. 2021 Jul 30;17:85. doi: 10.1186/s13007-021-00785-8 (PMC8325263; doi:10.1186/s13007-021-00785-8)
Supplement: Supplementary file 2 — Additional file 2: Fig. S1. Scatter plot for performance and heterosis of seedling BRTs versus heterozygosity. (a) Relationships between performance of seedling BRTs with heterozygosity. (b) Relationships between MPH of seedling BRTs with heterozygosity. Fig. S2. Prediction accuracies of seedling BRTs based on different numbers of markers. Corresponding numbers of markers were sampled randomly from 1631 markers five times to represent five repeats of the marker group. The mean of the prediction accuracies with 1000 runs for each repeat was plotted. [file 13007_2021_785_MOESM2_ESM.docx]

**Additional file 2**


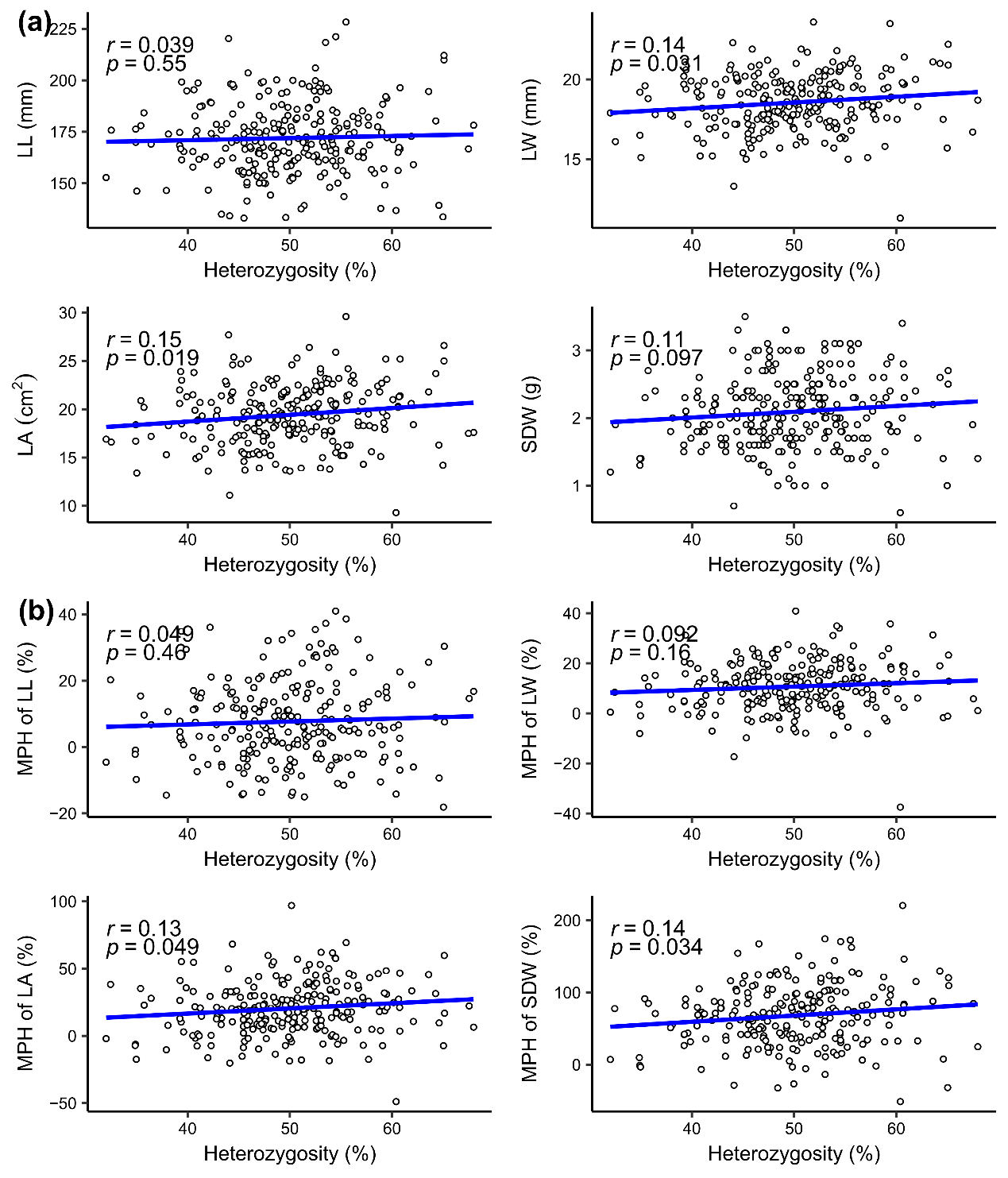


**Fig. S1.** Scatter plot for performance and heterosis of seedling BRTs versus heterozygosity. (a) Relationships between performance of seedling BRTs with heterozygosity. (b) Relationships between MPH of seedling BRTs with heterozygosity.


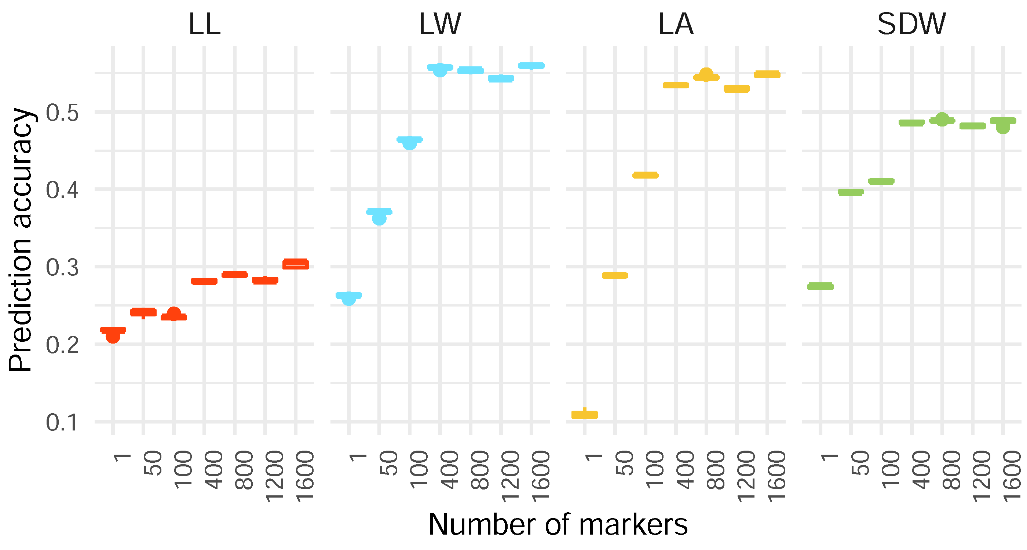


**Fig. S2.** Prediction accuracies of seedling BRTs based on different numbers of markers. Corresponding numbers of markers were sampled randomly from 1,631 markers five times to represent five repeats of the marker group. The mean of the prediction accuracies with 1,000 runs for each repeat was plotted.
